# Spatial distribution of uranium in mice kidneys detected by laser ablation inductively coupled plasma mass spectrometry

**DOI:** 10.17145/jab.17.007

**Published:** 2017-06

**Authors:** Venessa Jim, Corinne LaViolette, Margaret M. Briehl, Jani C. Ingram

**Affiliations:** 1 Department of Chemistry & Biochemistry, Northern Arizona University, Flagstaff, USA; 2 Department of Pathology, University of Arizona, Tucson, USA

**Keywords:** uranium, microprobe, mice kidney, LA-ICP-MS

## Abstract

The aim of the study is to better understand where uranium deposits in mice kidneys. The spatial distribution of uranium was examined in the kidneys of C57BL/6 mice using laser ablation inductively coupled plasma mass spectrometry (LA-ICP-MS). Mice were exposed to varying levels of uranyl nitrate in their drinking water. Calibration standards were developed to allow for semi-quantitative measurement of uranium in the cortical and medullary regions of mice kidney by LA-ICP-MS. Scanning electron microscopy was used to image the ablation patterns on the kidney. Uranium levels were observed to increase in kidney tissue as uranyl nitrate treatment exposure levels increased. A trend towards a higher uranium concentration in the medullary versus cortical region of the kidneys was observed. These results show the usefulness of LA-ICP-MS in toxicity studies by providing a quantitative, spatial assessment of uranium deposition in a target organ.

## Introduction

The bio-imaging of elements within biological tissue is important for studying diseases mediated by essential and toxic metals. The kidney is recognized as a major site for uranium accumulation [1–3]. Potential functional and structural targets of uranium toxicity include cell types involved in filtration (glomeruli and a tubule system) and in concentration of the urine (collecting tubules and ducts). Glomeruli are located in the cortex, or outer layer, of the kidney. The tubule system begins with the proximal convoluted tubule adjacent to the glomerulus. It then descends into the medullary, or inner portion of the kidney before looping back into the cortex to connect with the collecting tubule. Different cell types that vary in their absorptive properties are found along the length of the kidney’s filtration and collecting tubules systems. The distribution of uranium accumulation in the cortex and medulla of the kidney could provide mechanistic insights to its toxicity.

Rats and mice exposed to uranium compounds by injection, inhalation or through drinking water, show signs of nephrotoxicity [4]. Studies of histological changes indicate the most damage in the corticomedullary region containing the pars recta segment of the nephron [5–8]. Previous analyses of uranium toxicity in the kidney are limited, however, by the lack of quantitative measurements of this element in different regions of the kidney. Laser ablation inductively coupled plasma mass spectrometry (LA-ICP-MS) has been developed as an analytical technique for directly measuring trace and ultra-trace concentrations of elements at important sites within an organ [9–11]. One challenge to microprobe analysis of biological tissue using LA-ICP-MS is developing matrix matched standards for calibration [12,13]. The work reported here was focused on using LA-ICP-MS to semi-quantitatively determine the spatial distribution of uranium in the kidneys from mice exposed to varying levels of uranium in their drinking water.

## Materials and Methods

### Uranium Exposure Treatments and Preparation of Mice Tissue

The experimental protocol was approved by the University of Arizona’s Institutional Animal Care and Use Committee prior to commencement of the study and conformed to the National Institute of Health’s guidelines. Male (n=24) and female (n=24) C57BL/6 strain mice at five weeks of age were obtained from Taconic Farms (Germantown, NY). After a two week acclimation period in the animal care facility, each sex was divided into six treatment groups with four animals per group. A control group received water provided by the animal care facility at the University of Arizona (no added uranyl nitrate); the remaining groups received a constant supply of water to which uranyl nitrate hexahydrate, UO_2_(NO_3_)_2_ • 6 H_2_O was added to give final concentrations of 0.02, 0.2, 2.0, 20, or 200 mg/L U; this was the sole water source provided to the treated mice. The 0.02 mg/L level represents a concentration of uranium somewhat lower than the maximum contaminant level (MCL) established by the Environmental Protection Agency (EPA); the MCL for uranium is 0.03 mg/L [14]. After 30 days, the animals were euthanized; organs were harvested, frozen in liquid nitrogen, and stored at −70 °C. Tissue samples were embedded in an optimum cutting temperature compound (OCT) (Tissue Tek, Sakura Finetek Lot # 4583) in preparation for LA-ICP-MS analyses. The OCT was used to line the bottom of plastic embedding molds, which were then frozen. The kidneys were placed on the frozen OCT and oriented to produce coronal sections when cut with a cryostat microtome. Coronal sections provide the greatest surface area of the cortical and medullary regions [15]. The kidneys were covered with OCT, frozen and stored at −80 °C for 2 weeks. The samples were transported on dry ice to the cryostat microtome and allowed to reach a temperature of −21°C. The samples were sectioned with Leica 818 high profile microtome blades in 25 µm slices and mounted on plastic slides.

### Bulk Uranium Analysis

Quantification of uranium in the mice kidneys from each treatment group were analyzed both as bulk and microprobe measurements. For each mouse, there was one pair of kidneys; uranium was measured in bulk using ICP-MS with one whole kidney and the other kidney was microprobed by LA-ICP-MS. For bulk analysis, the kidneys were ashed using a Thermo Lindberg/Blue M box furnace, acid digested with ultrapure nitric acid, and analyzed by ICP-MS for uranium. The uranium concentration per mass of dry kidney tissue weight was determined to be in the parts per trillion and billion range. Bulk analysis of kidneys in the treatment groups of the 0.02, 0.2 and 2 mg/L U are not reported due to the minimal kidney tissue available for analysis and the very low concentration observed which were similar to the uranium concentration of the control.

### Preparation of Calibration Standards

Stock solutions of uranyl nitrate at concentrations of 1000 mg/L (Peak Performance CRM Single-Element Uranium Standard, Lot # 09J031) and 10 mg/L (SPEX CertiPrep CRM, Claritas PPT grade uranium, Lot # CL4-103U) were used as the uranium primary standard for preparation of calibration standards.

Chicken livers (Sanderson’s Farms) were purchased from a local grocery store and processed the same day. The lobes were separated and external connective tissue, adipose tissue, and arteries were removed. The lobes were homogenized twice with a 30 mL Wheaton homogenizer and Teflon pestle, resulting in 30–50 mL of homogenate, which was stored at −70 °C. Within three days, the homogenate was used to prepare calibration standards. The frozen sample was allowed to reach room temperature and mixed thoroughly by vortexing for one minute. Approximately 1 g portions of homogenate were placed into 1.5 mL tubes and spiked with uranyl nitrate to obtain concentrations of uranium (ng) per wet mass of chicken liver (g) at 0, 5, 10, 50, 500, and 1000 ng/g. Each mixture was vortexed for 30 seconds, placed in 0.5 × 0.5 cm plastic biopsy cryomold and frozen with liquid nitrogen and stored in a −70 °C freezer. The calibration standards were mounted in the OCT compound and sectioned using a Reichert Jung Frigocut 2800N Cryostat Microtome at thicknesses between 5 µm and 25 µm. The sections were mounted on plastic microscope slides and stored at room temperature until analysis.

### LA-ICP-MS Instrumentation and Analysis

Analysis was accomplished utilizing a New Wave UP-213 laser ablation system interfaced with a Thermo X Series II ICP-MS at Northern Arizona University. Laser and ICP-MS parameters were optimized. Ablation was done at 60% energy, with a repetition rate of 10Hz and a spot size of 30 µm. The line scan was utilized instead of a raster pattern in order to minimize ablation of the voided areas that were inescapable with raster patterns. The line pattern was unique for each calibration standard and mice kidney due to size differences and circumventing the void areas. The ICP-MS forward power was set to 1400 W, while the nebulizer gas (argon) was set to 0.60 L/min. Carrier gas flow (helium) from the laser to the ICP-MS was set to 800 mL/min. A NIST SRM 612 (Trace Elements in glass) glass standard was used to fine tune the LA-ICP-MS system before analysis of calibration standards and mice tissue samples. At the beginning of each experiment, the instrument was tuned for uranium at mass 238 because all signal reported in this work was for the uranium 238 isotope.

The sites of ablation were approximated for each kidney due to size differences, which ranged from 0.3–0.5 g. The cortical (edge) region was ablated at approximately 400–500 µm from the surface of the kidney. The medullary (center) ablation spanned the whole inner region of the kidney and was > 700 µm from the edge scans. The area of ablation was at least 33,000 µm^2^ for each pattern. A total of six ablation patterns were made along the edge of the kidney and another six ablation patterns were made through the center region of each kidney. The images of the ablated kidneys were taken with the digital camera attached to the laser ablation system. Additionally, the ablated kidneys were imaged by scanning electron microscopy to ensure complete ablation of the tissue. Uranium concentration was calculated with a calibration curve for the ablated tissue of the six scans along the edge of the kidney as well as the six scans through the center of the kidney. Each scan represented a signal average of four data collections within a 2.5 sec collection time. The results are reported as an average of the data collected from one kidney each from two different mice that had received the same concentration of uranyl nitrate spiked drinking water. Thus, the results are an average of a total of 12 scans and denoted as uranium concentration at the edge and center of the kidney, respectively. The uranium concentration at the edge and center were compared using Student’s t-test and the level of significance was set at *p<0.05*. All the data were reported as mean ± standard error of the mean. The means of the control group versus the exposed mice were analyzed for significant differences by one-way analysis of variance (ANOVA) and level of significance was set at *p<0.05*.

## Results

Quantification of uranium in the mice kidneys from each treatment group were analyzed both as bulk and microprobe measurements. Bulk analysis of the kidney was done to confirm that exposure to increasing levels in the drinking water resulted in accumulation of uranium in the mice kidney. Bulk analysis of kidneys in the treatment groups of the 0.02, 0.2 and 2 mg/L U are not reported due to the very low concentration observed which were similar to the uranium concentration of the control. The uranium concentration for the control based on an average of one kidney each from two different mice was 0.07 (± 0.02) ng/g. Similarly, using one kidney each from two different mice, the uranium concentration for the 20 mg/L U treatment group was determined to be 1.2 (± 0.8) ng/g and for the 200 mg/L U treated kidneys was determined to be 6.8 (± 3.3) ng/g. These results indicate that there is an increasing accumulation of uranium in the kidneys with increasing exposure to uranium in the mice drinking water.

Laboratory calibration standards were developed and tested for accuracy with LA-ICP-MS. Chicken livers were chosen as the matrix for the calibration standards based on ease of homogenization, availability of this tissue and the previous use of chicken tissue as standards when measuring uranium content in tissues [16]. An external calibration approach was taken in this work which consisted of utilizing chicken livers spiked with known concentrations of uranium. Internal standards were explored for this project; however, the elements assessed for use as an internal standard were not satisfactory due to signal variability. Thus, the calibration approach used here is considered semi-quantitative [17]. The calibration plot used eight laboratory-developed standards, ranging in concentration from 0 to 1000 ng/g uranium. A least square’s fit was used to determine the line equation best representing the calibration plot (*y=9.57x+1.14*) and the square of the correlation coefficient (R^2^ value) was determined to be 0.991 (data not shown). The average uranium signal values for each calibration standard were averaged for four analyses. The precision was evaluated based on the percent relative standard deviation for these averaged values, which ranged from 3 to 13%. For each separate analysis of the standard, the laser beam ablated a different location of the tissue surface. It was found that grinding the chicken liver samples twice increased the homogeneity of spiked standards, which resulted in improved precision.

**Figure 1:**
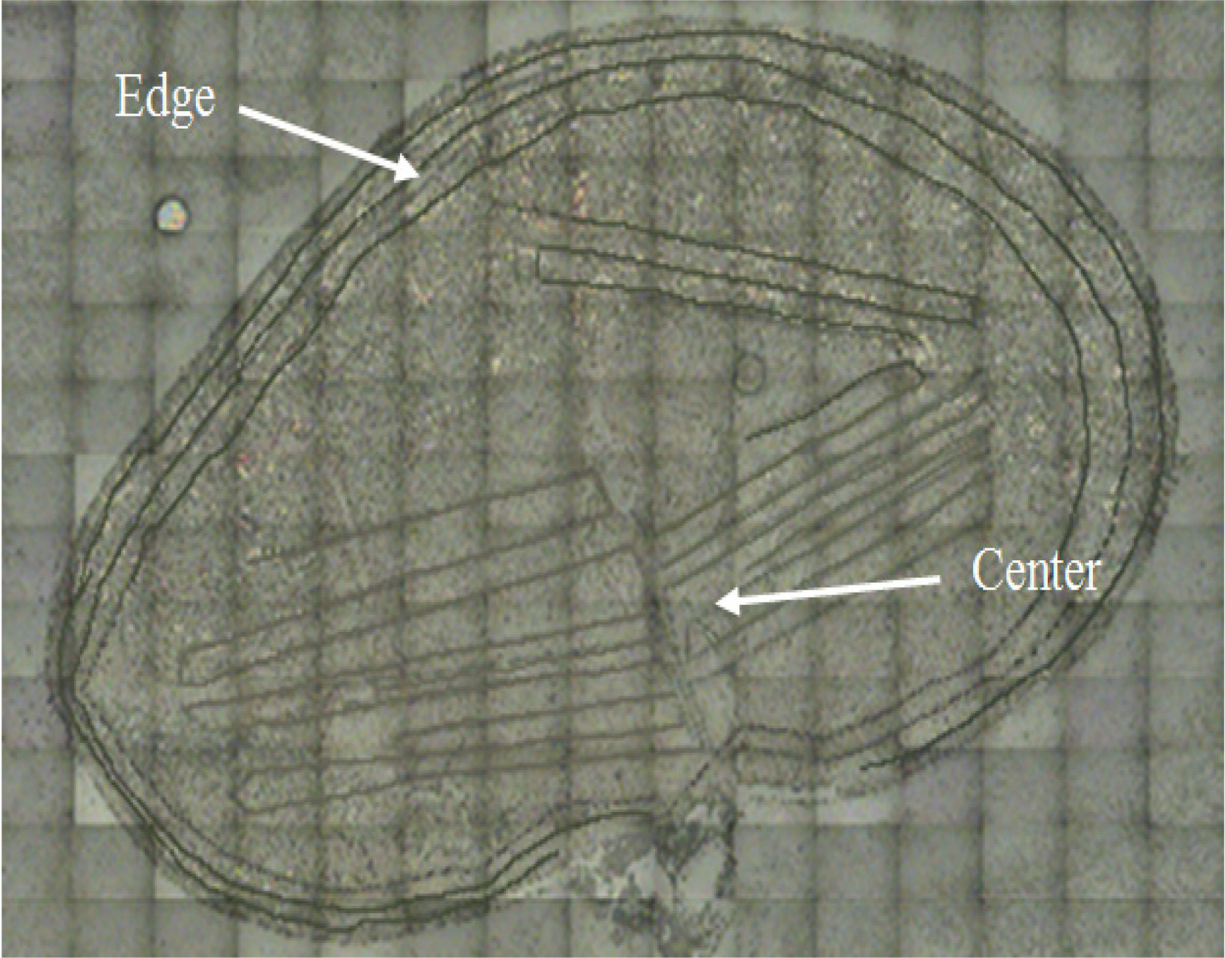
Scanning electron microscopy image of kidney from the control treatment group; ablation patterns are shown at edge and center of mice kidney. The size of the image is 9838 µm × 6888 µm.

**Figure 2:**
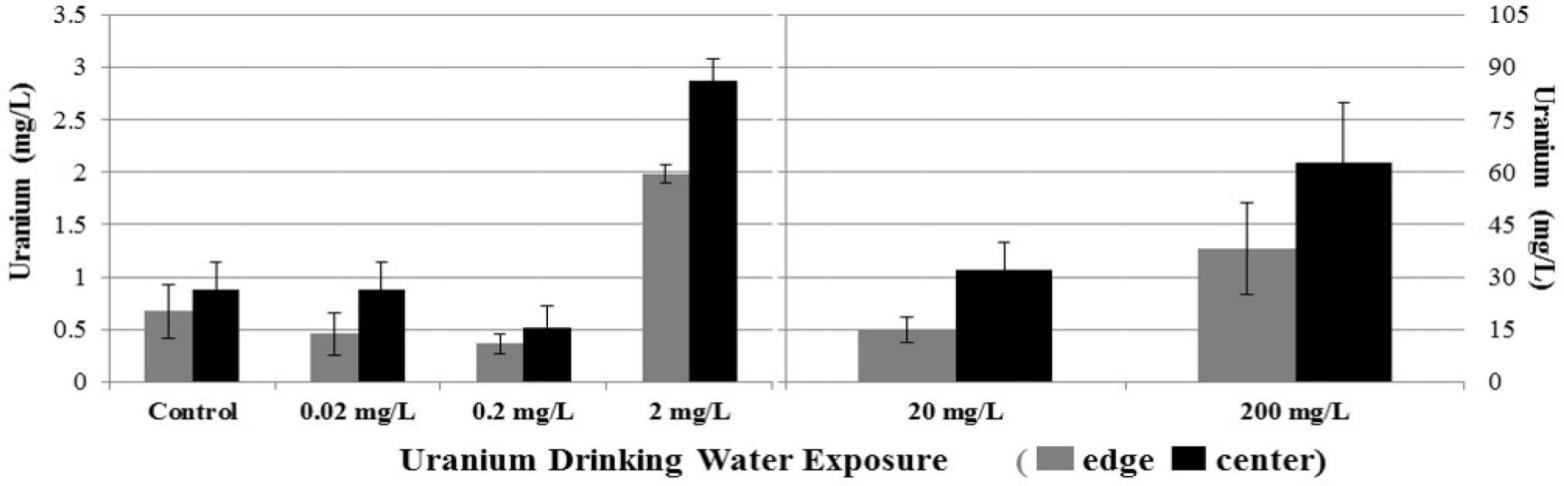
Uranium concentrations at the edge and center of mice kidney for mice exposed to 0 to 200 mg/L uranyl nitrate added to their drinking water. Results represent the average ± the standard deviation measured in a kidney from two mice with six scans per region per kidney (n=12). The U concentration at the edge and center were compared using Student’s t-test and the level of significance was set at *p<0.05*. Significant differences between the center and edge were observed for the 2, 20, and 200 mg/L

The mice kidneys that had been exposed to varying concentrations of uranyl nitrate in the mice drinking water (corresponding to 0 to 200 mg/L added uranium) were analyzed by LA-ICP-MS; quantitation was achieved using the calibration plot described above. Measurements of uranium concentration were taken at the edges and centers of mice kidneys were compared (Figure 1). The amount of uranium measured in the kidney tissue increased significantly for mice in the treatment groups given 2 to 200 mg/L U as compared to mice in the control group and lower treatment levels (Figure 2). In the 2 to 200 mg/L U treatment groups, uranium was observed to accumulate at greater concentrations in the center as compared to the edge regions of the kidney tissue (Figure 2). For example, in the 20 mg/L U treatment group, the average uranium concentration at the center and edge were 32 ± 7.6 and 15 ± 3.8 µg/L, respectively. This difference is significant (*p<0.05*). In contrast, the lower uranyl nitrate treatment groups (control to 0.2 mg/L U) showed no significant difference in uranium accumulation between the edge and center regions of the kidney. Overall, the results indicate that when there is a significant accumulation of uranium in the kidney, there is a particular spatial distribution of uranium between the edge and center regions of the kidney.

## Discussion and Conclusion

In this study, the application of LA-ICP-MS was used to characterize the spatial distribution of uranium accumulated in mice kidney as a result of chronic exposure to uranium through drinking water. Previous approaches to examining where uranium accumulates in the kidney include characterization using a combination of particle-induced X-ray emission (PIXE) and synchrotron radiation X-ray fluorescence (SXRF) [15] as well as secondary ion mass spectrometry (SIMS) microscopy [18]. The use of PIXE and SXRF is formidable as it provides sensitivity near ppm levels as well as spatial resolution as low as 0.2 µm; however, the major drawback of this approach is the use of a synchrotron source, which is not readily available to all researchers. SIMS microscopy provides spatial resolution as low as 0.5 µm and molecular as well as elemental detection with excellent sensitivity depending on the species. A major disadvantage in SIMS is the need for the sample to be under high vacuum conditions, which can result in disruption of biological samples. Reviews on the use of LA-ICP-MS for probing the spatial distribution of trace elements in biological tissue [9–11] describe the effectiveness of this approach. Since samples analyzed by LA-ICP-MS remain at atmospheric conditions, the physiological structure of the sample can be maintained throughout the analysis. The results shown here demonstrate the high elemental sensitivity as well as quantitation of spatial distribution as strengths of the LA-ICP-MS approach.

In prior studies, elemental imaging by PIXE and SXRF showed that in rats exposed to a single subcutaneous injection of uranyl acetate, the uranium accumulates in the pars recta section of the proximal tubule located in the corticomedullary region [15]. Injury in this region is indicated by increased numbers of apoptotic cells. The results are consistent with some histological assessments of kidney damage after acute exposure of rats [19] or mice [7] to uranyl fluoride or uranyl nitrate. Tessier et al. [18] employed SIMS microscopy to study uranium deposition in rats chronically exposed to uranyl nitrate through the drinking water. For up to 12 months of exposure, uranium accumulation was primarily in the proximal convoluted tubule. After 18 months, the distribution extended to more distal regions of the nephron. These assessments were qualitative, however, and did not provide measurements of uranium content. Other studies have reported tubular damage involving both the cortex and medulla of kidneys in mice injected with uranyl nitrate [8] or uranyl acetate [6] or in rats implanted with depleted uranium [20].

Our results extend the previous studies by providing a quantitative comparison of uranium content in different regions of the kidney. They are consistent with uranium accumulation throughout the kidney after chronic ingestion of uranyl nitrate by mice. The trend towards a higher concentration in the medullary versus cortical regions points to the ascending and descending loops of Henle and the collecting ducts as possible targets of uranium-mediated nephrotoxicity. Future studies could determine the extent to which chronic uranium exposure damages specific cells in these structures to impair renal function. Using mice as the model system facilitates a genetic approach to testing potential mechanisms of toxicity in regions where this element accumulates. Finally, our study validates LA-ICP-MS as a quantitative approach to assessing the spatial distribution of uranium in organs and tissues following long-term exposure to this environmental toxin.
